# Selective image segmentation driven by region, edge and saliency functions

**DOI:** 10.1371/journal.pone.0294789

**Published:** 2023-12-15

**Authors:** Shafiullah Soomro, Asim Niaz, Toufique Ahmed Soomro, Jin Kim, Adnan Manzoor, Kwang Nam Choi

**Affiliations:** 1 Department of Computer Science and Engineering, Chung-Ang University, Seoul, Republic of Korea; 2 School of Computing and Mathematics, Charles Sturt University, Bathurst, Australia; 3 SecuLayer Inc., Seoul, South Korea; 4 Department of Artificial Intelligence, Quaid-e-Awam University of Engineering Science and Technology, Nawabshah, Sindh, Pakistan; 5 Department of Computer Science and Media Technology, Linnaeus University, Vaxjo, Sweden; Vellore Institute of Technology, INDIA

## Abstract

Present active contour methods often struggle with the segmentation of regions displaying variations in texture, color, or intensity a phenomenon referred to as inhomogeneities. These limitation impairs their ability to precisely distinguish and outline diverse components within an image. Further some of these methods employ intricate mathematical formulations for energy minimization. Such complexity introduces computational sluggishness, making these methods unsuitable for tasks requiring real-time processing or rapid segmentation. Moreover, these methods are susceptible to being trapped in energy configurations corresponding to local minimum points. Consequently, the segmentation process fails to converge to the desired outcome. Additionally, the efficacy of these methods diminishes when confronted with regions exhibiting weak or subtle boundaries. To address these limitations comprehensively, our proposed approach introduces a fresh paradigm for image segmentation through the synchronization of region-based, edge-based, and saliency-based segmentation techniques. Initially, we adapt an intensity edge term based on the zero crossing feature detector (ZCD), which is used to highlight significant edges of an image. Secondly, a saliency function is formulated to detect salient regions from an image. We have also included a globally tuned region based SPF (signed pressure force) term to move contour away and capture homogeneous regions. ZCD, saliency and global SPF are jointly incorporated with some scaled value for the level set evolution to develop an effective image segmentation model. In addition, proposed method is capable to perform selective object segmentation, which enables us to choose any single or multiple objects inside an image. Saliency function and ZCD detector are considered feature enhancement tools, which are used to get important features of an image, so this method has a solid capacity to segment nature images (homogeneous or inhomogeneous) precisely. Finally, the adaption of the Gaussian kernel removes the need of any penalization term for level set reinitialization. Experimental results will exhibit the efficiency of the proposed method.

## Introduction

Image segmentation plays a pivotal role in image processing and computer vision, serving as a crucial step for various applications, such as image recognition and object detection. Over the last decade, numerous methods have emerged for image segmentation, encompassing techniques like region merging, thresholding-based methods, edge detection approaches, as well as clustering-based methods. Active contours, pioneered by Kass [1], have found widespread application in image segmentation, with subsequent adaptations introduced by various researchers [2–4].

The core concept of active contour is to guide the curve’s motion toward the object boundary using energy minimization algorithms. The initial structure of active contours, however, exhibited two distinct drawbacks. Firstly, it was highly sensitive to its starting position. Secondly, managing topological changes that arose during the curve’s evolution posed a challenge. Addressing this concern, Osher and Sethian [5] introduced a curve model for the moving front. In this model, the curve was implicitly represented by a zero level set function. Subsequently, a multitude of variations on the level set models emerged, which could be categorized into two primary classes: edge-based and region-based models. Edge-based models [1, 6] operate by responding to the image’s gradient to achieve image segmentation. These approaches enable level set segmentation without the need for re-initialization. Nonetheless, edge-based models often encounter challenges when dealing with delicate boundaries, making accurate identification problematic.

Region-based models aim to identify distinct regions by utilizing specific descriptors, such as color or intensity, to guide contour movement. Recently, these models have gained significant attention for their ability to capture regional data [7–21]. Initially designed to segment objects with uniform intensity, traditional region-based models, like the Chan-Vese (CV) model [8], excel at capturing images with similar intensity levels. However, the CV model struggles when faced with intensity variations or texture information in images. To address this, region-based models have been enhanced and adapted into local region-based methods for segmenting images with inhomogeneous intensity variation [10]. Notably, Li et al. introduced the first local fitting model, known as LBF [10]. This model employs the concept of region scalable fitting energy (RSF) to capture inhomogeneous intensity patterns, facilitated by local adaptation using Gaussian kernels.

Similarly, in the context of addressing local energy considerations, the VLBCS (Variational Level Set Approach for Bias Correction and Segmentation) method, as described in [22], and the LSACM (Local Statistical Active Contour Model) approach, as presented in [23], were introduced. These techniques have further improved the outcomes of image segmentation for cases involving inhomogeneous images by integrating bias field correction into their formulations. This integration allows them to approximate the distribution of inhomogeneities across the entire image [24].

Additionally, the LGDF (Local Gaussian Distribution Fitting) method, as detailed in [25], and the LSD (Local Signed Difference Energy) approach, as outlined in [26], were proposed. These methods incorporate considerations of local intensity into the evolution of the level set and have shown promising results when dealing with images exhibiting intensity inhomogeneities. However, it’s important to note that these methods come with notable limitations, particularly concerning the need for level set re-initialization and computationally complex formulations.

Recently, there has been a trend in reconfiguring edge-based models by substituting their edge term with a region term in order to construct the Signed Pressure Force (SPF) function. Among these approaches, one of the most noteworthy is the work of Zhang et al. as detailed in [9]. They were the first to introduce an SPF function that effectively captures regions in an adaptive manner. Subsequently, several techniques, as discussed in [15], have emerged in the field of image segmentation, focusing on both local and global SPF formulations for homogeneous and inhomogeneous images.

Recent research in cognitive psychology, neurobiology, computer vision, and image processing has revealed a strong connection between saliency detection and the human visual system. Consequently, numerous investigators have made efforts to incorporate saliency features into image segmentation processes, as demonstrated in previous studies such as those by Bai and Wang in [27], Qin et al. in [28], and Zhi et al. in [29].

For instance, Bai and Wang introduced the Saliency-SVM model in [27], which takes into account saliency data and formulates the image segmentation problem accordingly. In a similar vein, Qin et al., as described in [28], combined region and affinity propagation clustering algorithms with region saliency to develop a random walk-based image segmentation model. More recently, Zhi et al., detailed in [29], devised an active contour method based on regions and edges, incorporating saliency knowledge into the energy functional to enhance the segmentation process.

Real-world images often exhibit variations in intensity, color, and texture, presenting a significant challenge for traditional segmentation methods in effectively delineating distinct regions. This challenge becomes particularly pronounced when dealing with images that contain objects demonstrating both homogeneous and inhomogeneous characteristics. Additionally, certain existing approaches rely on complex mathematical formulations, which can result in sluggish computational performance during the energy minimization process. Our objective is to develop a method that strikes a balance between precision and computational efficiency, rendering it suitable for practical applications.

Furthermore, achieving precise boundaries for image regions, especially when dealing with objects characterized by faint or subtle edges, poses a substantial challenge for many segmentation techniques. Our aim is to devise an approach capable of accurately capturing these boundaries, even in such intricate cases. Moreover, different regions within an image may necessitate distinct segmentation strategies. Therefore, we endeavor to create a method that seamlessly integrates both local and global information to adaptively capture diverse image structures.

In our investigations, we have also observed that saliency detection, inspired by human visual perception, has yielded promising results in enhancing image comprehension and segmentation. This observation motivates us to leverage saliency information as a means to improve the accuracy and robustness of our segmentation method.

Guided by these motivations, our newly proposed active contour method has been meticulously crafted to tackle these formidable challenges head-on. Our method leverages a combination of key components, including a zero-crossing detector (ZCD) to extract edge information, saliency detection to discern the significance of regions, and a globally tuned region-based signed pressure force (SPF) term. Through this work, we strive to contribute a method that not only advances the field of image segmentation but also has practical implications for a wide range of applications.

This research proposes a novel active contour method with the following major contributions.

1. First this method captures the global structures of an image by using zero crossing feature detector and using it as a driving force for contour evolution.
2. This method has designed a new energy term by amalgamating saliency map function in level set formulation.
3. Proposed method can segment an inhomogeneous and homogeneous set of images by considering global SPF, ZCD and saliency information at some scaled value.
4. Proposed method is using a selective level set formulation from Zhang et al [9], this property allows the proposed method to capture the region of interest inside an image.

## Proposed model

As discussed earlier, active contours represent curve *C* by zero level set function as: *C* = (*x*, *y*)|*ϕ*(*x*, *y*) = 0 and describe level set energy functional as:

$$\begin{array}{c} \frac{d\phi }{dt}+F\left|\nabla \phi \right|=0 \end{array}$$ (1)

The force *F* in above energy function relies on image data and level set function *ϕ*.

Motivated by the work of [9, 29] methods, we propose a novel homogeneous and inhomogeneous image segmentation method by combining global SPF and ZCD terms with saliency function at some scaled value. The Proposed formulation of the saliency, region and ZCD based *E*_*SRZCD*_ method is defined as:

$$\begin{array}{c} \begin{array}{c} F_{SRZCD}\left(\phi \right)=s(w*F_{ZCD})+\left(1-w\right)(F_{SPF})+\left(1-s\right)\left(F_{SAL}\right) \end{array} \end{array}$$ (2)

Equation above is scaled with a parameter *s* which ranges between 0 ≤ *s* ≤ 1. This parameter evaluate the effect of the intensity and Saliency terms based on the image we have. We set *s* large when we want the intensity term as a leading force to capture homogeneous and inhomogeneous images with less saliency information. Similarly, we choose *s* small if we want saliency as a leading force to capture textual features from an image.

In Eq 2, *w* is also an important positive parameter ranges between 0 ≤ *w* ≤ 1, and it plays a key part in dealing with an image region and edge information. When *w* is close to 0 then the proposed energy work as a leading global segmentation method with some saliency information. Likewise, when *w* it is close to 1 then it has a predominant ZCD function which makes the proposed method act as an inhomogeneous image segmentation method with some saliency information.

Many researchers have previously employed various zero-crossing detectors to extract edge information. However, in our proposed energy function, we utilize the Difference of Gaussian (DoG) detector as a force term, denoted as *F*_*DoG*_, for detecting image boundaries. This method highlights regions where intensity changes rapidly, effectively serving as a zero-crossing edge detector. Additionally, we incorporate a DoG force term, *F*_*DoG*_, as an image enhancement feature. This feature is derived by subtracting a blurred version of the original image from a less blurred version of the same original image. It enhances the visibility of edges and is employed as an energy source for image segmentation. For an image denoted as *I*(*x*, *y*): Ω ← *R*^2^, the DoG term in Eq (2) is defined as:

$$\begin{array}{c} \begin{array}{c} F_{DoG(x,y)}=[I\left(x\right)*\frac{1}{\sigma_{1}\sqrt{2\pi }}exp(-\frac{x^{2}}{2\sigma_{1}^{2}})]-[I\left(x\right)*\frac{1}{\sigma_{2}\sqrt{2\pi }}exp(-\frac{x^{2}}{2\sigma_{2}^{2}})] \end{array} \end{array}$$ (3)

where *σ*_1_ and *σ*_2_ are the standard deviations of the Gaussian filters, where *σ*_1_ < *σ*_2_. These parameters plays a crucial role in obtaining the result and removing unwanted noise from an image. For noisy images, the difference of *σ*_1_ < *σ*_2_ must be chosen high and for images with less noise *σ*_1_ < *σ*_2_ must be chosen small to retain image details properly.

Edge-based active contour methods rely solely on gradient-based information, which can lead to imprecise segmentation outcomes. Consequently, these models often become trapped in local minimums or struggle to accurately delineate fuzzy and noisy edges. Therefore, in Eq (2), our proposed approach incorporates a global region-based SPF (Signed Pressure Force) function inspired by [11]. This function utilizes the global region information within an image, guiding the level set flow to shift the contour away from boundaries and effectively capture homogeneous regions. The formulation of the SPF function is described as follows:

$$\begin{array}{c} \begin{array}{c} SPF\left(I\right)=\frac{I\left(x\right)-\left(j_{1}+j_{2}\right)/2}{max(|I\left(x\right)-j_{1}+j_{2}/2)|} \end{array} \end{array}$$ (4)

where *j*_1_ and *j*_2_, are the intensity mean values taken from CV model [8] defined as:

$$\begin{array}{c} \begin{array}{cc} & j_{1}=\frac{\int_{\Omega }I(x)H_{\varepsilon }\left(\phi \left(x\right)\right)dx}{\int_{\Omega }H_{\varepsilon }\left(\phi \left(x\right)\right)dx}\\ & j_{2}=\frac{\int_{\Omega }I(x)\left(1-H_{\varepsilon }\left(\phi \left(x\right)\right)\right)dx}{\int_{\Omega }(1-H_{\varepsilon }\left(\phi \left(x\right)\right))dx} \end{array} \end{array}$$ (5)

SPF function is very adaptive with respect to its position so that it can shrink or expand it movement if placed inside or outside of object. *H*_*ε*_(*ϕ*) Eq (8) and in Eq (5) is the Heaviside term written as:

$$\begin{array}{c} H_{\varepsilon }\left(\phi \right)=\frac{1}{2}(1+\frac{2}{\pi }\text{arctan}(\frac{\phi }{\varepsilon })) \end{array}$$ (6)

where *ϵ* stabilize the smoothness of Heaviside term.

Saliency function in Eq (2) used to capture color and texture feature of an image. For *I*(*x*, *y*): Ω ← *R*^2^, we take saliency function adapted as [27].

$$\begin{array}{c} \begin{array}{c} S\left(x,y\right)=|I_{m}\left(x,y\right)-I_{G}\left(x,y\right)| \end{array} \end{array}$$ (7)

Where *I*_*m*_ is mean pixel value of an image and *I*_*G*_ = *I*(*x*, *y*) * *G* is an image smoothed by Gaussian kernel. Subsequently, by taking the derivative of 7 with respect to *s*_1_, and *s*_2_ we get the corresponding values of *s*_1_ and *s*_2_ inside Ω_*i*_*n* and outside Ω_*o*_*ut* as:

$$\begin{array}{c} \begin{array}{cc} & s_{1}=\frac{\int_{\Omega }S(x)H_{\varepsilon }\left(\phi \left(x\right)\right)dx}{\int_{\Omega }H_{\varepsilon }\left(\phi \left(x\right)\right)dx}\\ & s_{2}=\frac{\int_{\Omega }S(x)\left(1-H_{\varepsilon }\left(\phi \left(x\right)\right)\right)dx}{\int_{\Omega }(1-H_{\varepsilon }\left(\phi \left(x\right)\right))dx} \end{array} \end{array}$$ (8)

By looking at the adaptivity of the SPF function from [9], we have also redefined saliency function and formulated new adaptive saliency function as:

$$\begin{array}{c} \begin{array}{c} SAL\left(I\right)=\frac{s\left(x\right)-\left(s_{1}+s_{2}\right)/2}{max(|I\left(x\right)-s_{1}+s_{2}/2)|} \end{array} \end{array}$$ (9)

The traditional geodesic active contour (GAC) method [6] is considered as a pioneer active contour method, which uses an object gradient information from an edge. Let *I*: Ω ⊂ *R*^2^ is an image domain, *I*: Ω → *R* is an input image and *C(q)* is a closed curve. GAC has formulated the following energy formulation:

$$\begin{array}{c} E_{GAC}\left(C\left(q\right)\right)=\int_{0}^{1}h\left(|\nabla I\left(C\left(q\right)\right)|\right)C^{\prime }\left(q\right)dq, \end{array}$$ (10)

*h* is the edge stopping function designed as:

$$\begin{array}{c} h(\nabla I)=\frac{1}{1+|\nabla G_{\sigma }*I|} \end{array}$$ (11)

∇*G*_*σ*_ * *I* is known as the convolution of an image *I* with a kernel having standard deviation *σ*. We get the following formulation after minimizing above energy functional.

$$\begin{array}{c} C_{t}=h\left(|\nabla I|\right)\left(k+\alpha \right)\vec{N}-\left(\nabla h.\vec{N}\right)\vec{N} \end{array}$$ (12)

where *α* is included to increase the propagation, *k* is curvature of the curve and $\vec{N}$ is inward normal of the curve. The final level set equation is defined as follows:

$$\begin{array}{c} \frac{\partial \phi }{\partial t}=h\left|\nabla \phi \right|(div(\frac{\nabla \phi }{\left|\nabla \phi \right|})+\alpha )+\nabla h.\nabla \phi \end{array}$$ (13)

Finally, by substituting the *F*_*SMLOG*_ from 2 in to 13, the Gateaux derivative of the proposed method can be written as.

$$\begin{array}{c} \frac{\partial \phi }{\partial t}=F_{SMLOG}(I\left(x\right)(div(\frac{\nabla \phi }{\left|\nabla \phi \right|})+\alpha )+\nabla F_{SMLOG}(I\left(x\right).\nabla \phi \end{array}$$ (14)

It can be seen that the 1st term of the equation above is using some penalizing energy term to maintain the level set deviation from a signed distance function. However, the proposed method adopts instead a Gaussian kernel to regularize the level set function, which maintains the stability of the level set as a signed distance function. Therefore, we can eliminate the $div(\frac{\nabla \phi }{\left|\nabla \phi \right|})$ from 14. Moreover, we are also incorporating regional information of an image in our formulation, which avoids edge leakage problems occurring in only edge-based methods. In this context, we can also remove unnecessary term ∇*F*_*SMLOG*_(*I*(*x*).∇*ϕ* from 14. Therefore, the final level set equation is rewritten as:

$$\begin{array}{c} \frac{\partial \phi }{\partial t}=F_{SMLOG}\left(I\left(x\right).\alpha |\nabla \phi \right| \end{array}$$ (15)

The level set function in active contours is often required to be initiated as SDF (signed distance function) and also to be reinitialized to maintain its stability. However, the proposed method has deployed the Gaussian kernel after each iteration, which not only removes the problem of costly re-initialization the but also maintains the stability of the level set function. We define the initial level set function *ϕ*_0_ as:

$$\begin{array}{c} \phi \left(x,t=0\right)=\{\begin{array}{c} -\rho \;x\in \Omega \backslash \partial \Omega \\ 0\;x\in \partial \Omega \\ \rho \;x\in \Omega \backslash \Omega \end{array} \end{array}$$ (16)

*ρ* ≥ 0 is a constant value, Ω_0_ represents a region inside an initial contour, Ω is the image domain and ∂Ω is an initial contour. Finally in order to obtain the results of our proposed method, we follow the following iterative procedure:

**Algorithm**

1. **Initialize** level set as constant function, *ϕ*(*x*, *t* = 0) from Eq (16).

2. *n* = 1.

3. **while** the solution is not converged **do**

4. Compute the values of DoG, SPF and SAL function from (9), (4) and from (4).

5.  Solve the final level set from (15).

6.  *n* = *n* + 1.

7. **end while**

8. **Output**: Final result, *ϕ*.

## Results and quantitative comparisons

Each experiment of this paper executed over MATLAB 2018 on a Windows 10 in a PC with Intel i7, 2.9 GHz with 16 GB RAM. We have chosen the parameters for our experiments as *ρ* = 1, *ϵ* = 1.5, *σ* = 1, *σ*_1_ = 1, *σ*_2_ = 2, Δ*t* = 1 and *α* = varies for image to image.

**Fig 1:**
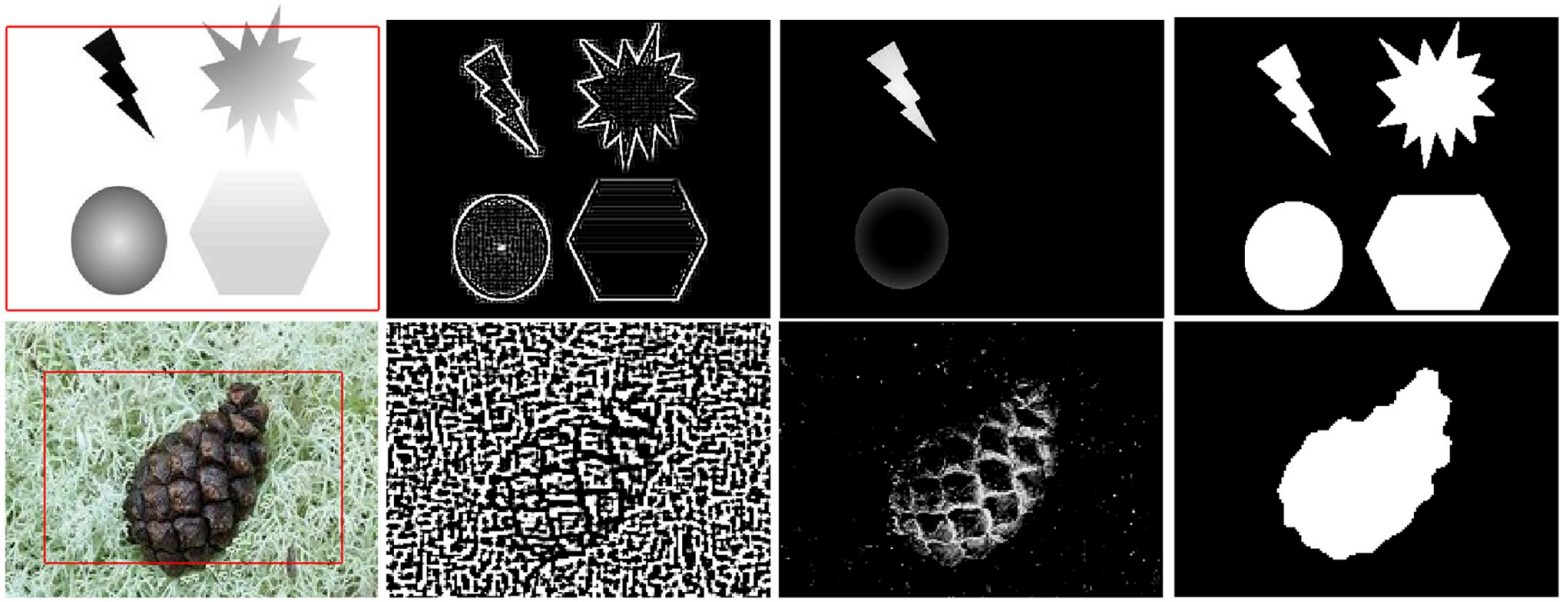
Proposed method outcomes for Inhomogeneous (1^*st*^ row) and real texture (2^*nd*^ row) image segmentation. Column I: Original images with initial level set, column II: DOG profile of an image, column III: Saliency profile of an image and column IV: Final segmentation result.

To demonstrate the effectiveness of our proposed method, we initially applied it to both grayscale intensity and real RGB images. The results generated by our approach can be seen in Fig 1. In 1^*st*^ row of Fig 1, we present the outcome obtained from the grayscale intensity image. Given that this image primarily consists of intensity regions, we set the parameters as follows: *σ*_1_ = 1, *σ*_2_ = 10, *w* = 0.9, and *s* = 0.999. These parameter settings prioritize the utilization of the Difference of Gaussians (DoG) and Signed Pressure Force (SPF) components while minimizing the emphasis on saliency information, resulting in a precise delineation of regions. Similarly, In the 2^*nd*^ of Fig 1, we display the results we obtained from a real RGB texture image. For this image, we adjusted the settings as follows: *σ*_1_ = 2, *σ*_2_ = 20, *w* = 0.01, and *s* = 0.01. These settings were chosen to improve the segmentation by giving more importance to the saliency force while reducing the influence of edge and region details.

**Fig 2:**
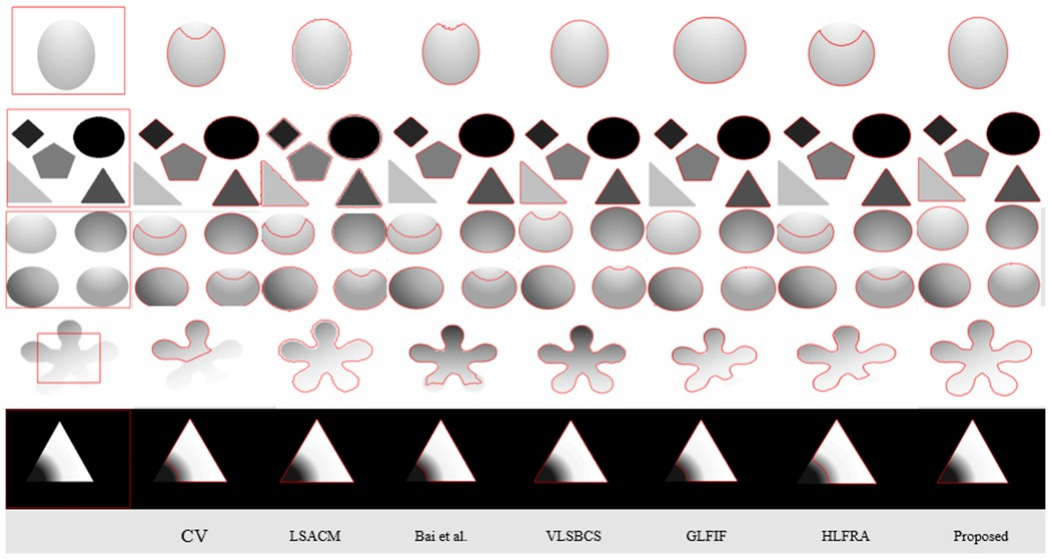
Proposed method comparision on images having intensity inhomogeneity. Column 1^*st*^: (Initial Position), Column 2^*nd*^: (CV [8]), Column 3^*rd*^: (LSACM [23]), Column 4^*th*^: (Bai et al. [27]), Column 5^*th*^: VLSBCS. [2], Column 6^*th*^: GLFIF. [30], Column 7^*th*^: HLFRA. [31] and Column 8^*th*^: (proposed method) respectively.

Further, we have shown intensity inhomogeneity analysis of five different images with other state of art methods in Fig 2. The results demonstrate that CV [8] is unable to handle inhomogeneity across the images and eventually failed to segment objects. Bai et al. [27] is using only saliency information, therefore it has also failed to get accurate results. As LSACM [23] and VLSBCS [2] are using local pixel information with bias intensity correction, therefore this method has got some positive outcomes to some extent. However, the proposed method has successfully segmented all the images accurately compared to all previous methods. Computational complexity of Fig 2 is compared in Table 1 in terms of time and iterations taken by each method. CV [8] method is very fast compared to other methods yet failed to get accurate outcome. Proposed method consumed a smaller number of iterations than other methods while getting accurate outcomes.

**Table 1:** CPU time comparison (in seconds) and iterations consumed by each method in Fig 2.

Methods		Image 1	Image 2	Image 3	Image 4	Image 5
CV [8]	Iterations	60	60	100	60	60
	CPU time(sec)	**3.564**	**3.142**	**3.721**	**4.0454**	**3.273**
LSACM [23]	Iterations	100	100	100	100	100
	CPU time(sec)	12.752	14.435	12.752	13.452	14.624
Bai et al. [27]	Iterations	30	30	30	30	30
	CPU time(sec)	3.324	3.745	3.954	3.541	3.965
VLSBCS. [2]	Iterations	100	150	150	150	150
	CPU time(sec)	9.354	11.784	12.745	10.452	3.365
GLFIF. [30]	Iterations	100	100	100	100	100
	CPU time(sec)	7.345	9.547	10.874	9.653	4.654
HLFRA. [31]	Iterations	100	150	150	150	80
	CPU time(sec)	6.156	10.745	10.456	9.354	4.474
Proposed method	Iterations	100	100	100	100	100
	CPU time(sec)	6.150	2.877	7.752	2.674	3.252

**Fig 3:**
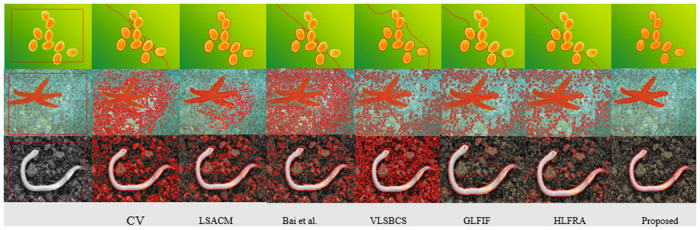
Proposed method comparision over real set of images. Column 1^*st*^: (Initial Position), Column 2^*nd*^: (CV [8]), Column 3^*rd*^: (LSACM [23]), Column 4^*th*^: (Bai et al. [27]), Column 5^*th*^: VLSBCS. [2], Column 6^*th*^: GLFIF. [30], Column 7^*th*^: HLFRA. [31] and Column 8^*th*^: (proposed method) respectively.

Subsequently, the result of the proposed method is obtained over the real set of images and compared with other methods in Fig 3. The results demonstrate that CV [8] is unable to handle real set of images and eventually failed to segment objects. Bai et al. [27] is using only saliency information, which is not sufficient to segment out an inhomogeneous real set of images. Moreover, LSACM [23] and VLSBCS [2] are using only local pixel information, which cannot capture texture details of images. on the other hand, the proposed method has successfully segmented all the images accurately compared to all previous methods. In Table 2, we have measured the time complexity and number of iterations of each method consumed over every single image in Fig 3,. The results have proved that the proposed method has consumed a smaller number of iterations and lesser time than other methods.

**Table 2:** CPU time comparison (in seconds) and iterations of previous methods and proposed method in Fig 3.

Methods		Image 1	Image 2	Image 3
CV [8]	Iterations	50	50	50
	CPU time(sec)	4.325	3.816	3.721
LSACM [23]	Iterations	200	200	200
	CPU time(sec)	15.725	16.374	14.574
Bai et al. [27]	Iterations	60	60	60
	CPU time(sec)	4.364	4.745	4.965
VLSBCS. [2]	Iterations	200	200	200
	CPU time(sec)	10.965	12.745	13.458
GLFIF. [30]	Iterations	100	100	100
	CPU time(sec)	8.6584	9.657	10.874
HLFRA. [31]	Iterations	100	100	100
	CPU time(sec)	9.987	10.457	10.975
Proposed method	Iterations	40	40	40
	CPU time(sec)	**3.324**	**3.245**	**3.142**

**Fig 4:**
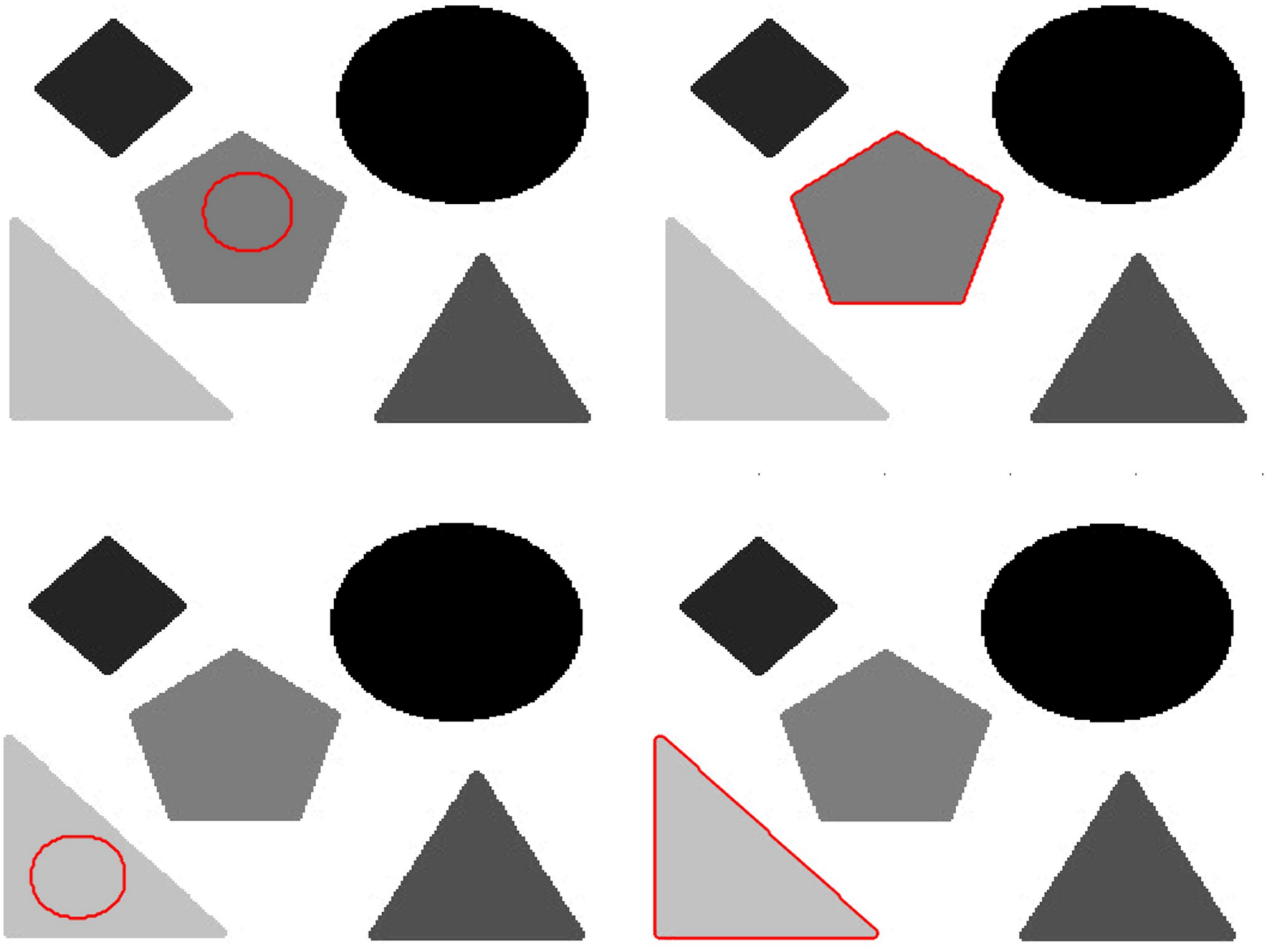
Selective segmentation result of the proposed method. Column 1^*st*^: (Initial Position), Column 2^*nd*^: (Selective outcome).

Fig 4 illustrates the selective segmentation property of the proposed method. The first column shows the selective initialization, whereas the second column demonstrate the selective segmentation result of the proposed method.

**Fig 5:**
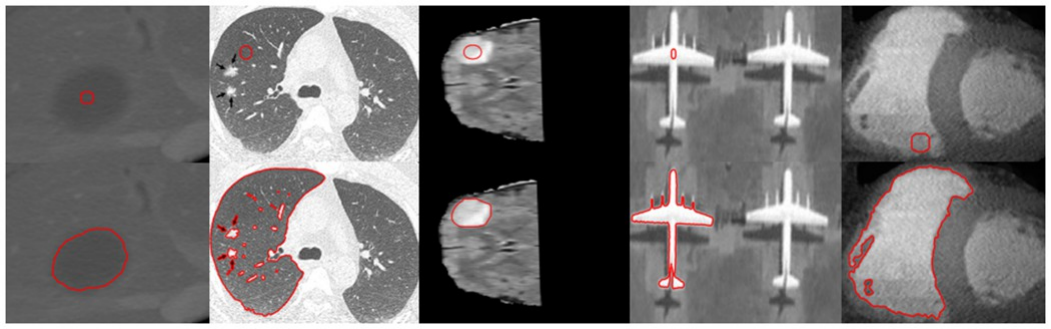
Segmentation results of the proposed method with region of interest. Row I: (Initial Position), Row II: (Result of the proposed method).

By taking an advantage of the selective property of the proposed method. In Fig 5, we have performed segmentation over five different images to capture the region of interest. The first row illustrates the initial position of the contour, which is placed inside of the region and the second row illustrates the corresponding outcomes. In the first column, we have used a liver tumor image taken from [32], the second column shows a brain tumor image taken from the BRATS 2015 dataset [33], the third column shows a lung CT image taken from the covid dataset [34], the fourth column shows a synthetic image and the last column shows a cardiac image taken from public repository.

**Fig 6:**
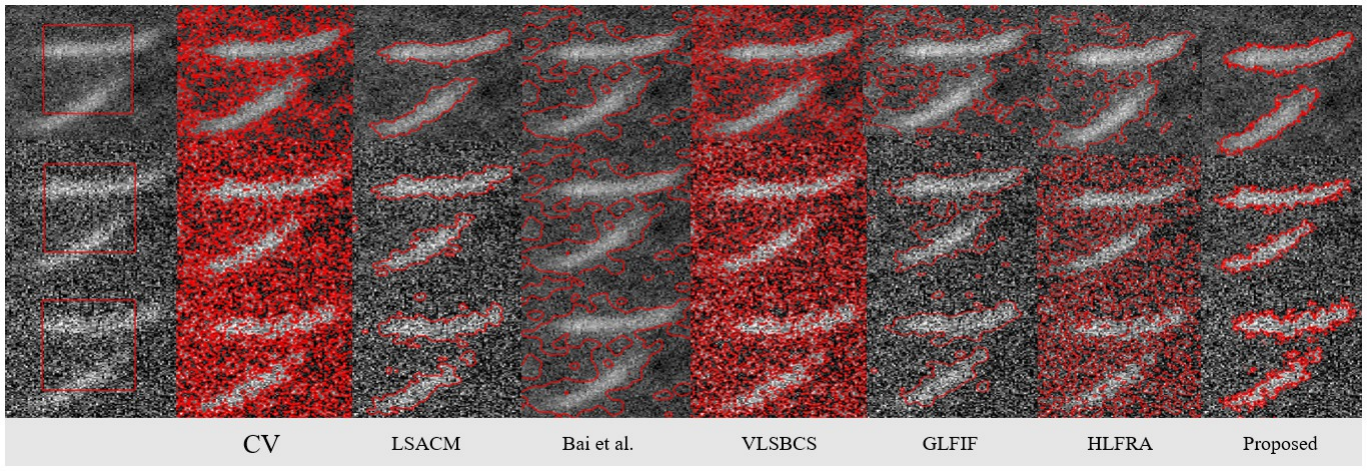
Proposed method comparision over noisy images. Column 1^*st*^: (Initial Position), Column 2^*nd*^: (CV [8]), Column 3^*rd*^: (LSACM [23]), Column 4^*th*^: (Bai et al. [27]), Column 5^*th*^: VLSBCS. [2], Column 6^*th*^: GLFIF. [30], Column 7^*th*^: HLFRA. [31] and Column 8^*th*^: (proposed method) respectively.

In Fig 6, we have performed the proposed method segmentation and its comparison with other methods over an image with varying noise details. The results have demonstrated that the proposed method has outperformed previous methods in terms of noisy image segmentation. Moreover, we have measured the time complexity and number of iterations of each method in Table 3. It is clearly illustrated that the proposed method has achieved the required results in less time and in a less number of iterations.

**Table 3:** CPU time comparison (in seconds) and iterations of previous methods and proposed method in Fig 6.

Methods		Image 1	Image 2	Image 3
CV [8]	Iterations	50	50	50
	CPU time(sec)	3.957	3.124	3.475
LSACM [23]	Iterations	200	200	200
	CPU time(sec)	25.254	26.785	26.954
Bai et al. [27]	Iterations	60	60	60
	CPU time(sec)	4.421	4.742	4.965
VLSBCS. [2]	Iterations	200	200	200
	CPU time(sec)	8.654	8.354	8.452
GLFIF. [30]	Iterations	100	100	100
	CPU time(sec)	6.548	6.758	7.654
HLFRA. [31]	Iterations	100	100	100
	CPU time(sec)	7.741	7.542	7.546
Proposed method	Iterations	50	50	50
	CPU time(sec)	**2.854**	**2.985**	**2.754**

**Fig 7:**
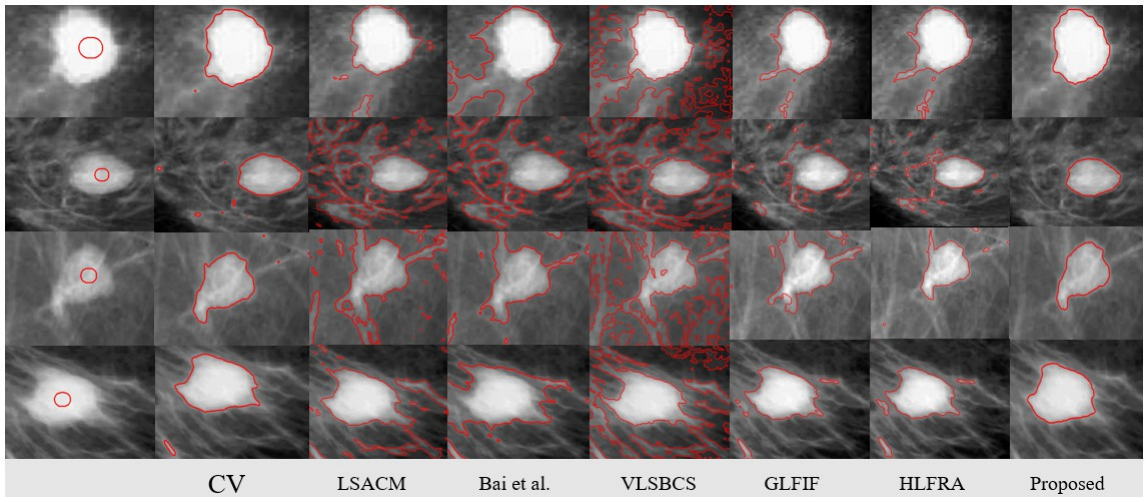
Proposed method comparision over mammogram tumor images. Column 1^*st*^: (Initial Position), Column 2^*nd*^: (CV [8]), Column 3^*rd*^: (LSACM [23]), Column 4^*th*^: (Bai et al. [27]), Column 5^*th*^: VLSBCS. [2], Column 6^*th*^: GLFIF. [30], Column 7^*th*^: HLFRA. [31] and Column 8^*th*^: (proposed method) respectively.

In Fig 7, we have taken mammogram images from mini-Mias [35] dataset to validate our method performance, the results have illustrated that all previous methods were not capable to capture the region of interest due to the deficit of characteristic function. Since, the proposed method is using the selective characteristic function, which can capture the desired region based on contour initialization. Therefore, the proposed methods have segmented all regions perfectly. We have also measured the time complexity and number of iterations of each method in Table 4. It shows that the proposed method has acquired segmentation results in minimum time and iterations.

**Table 4:** CPU time comparison (in seconds) and iterations of previous methods and proposed method in Fig 7.

Methods		Image 1	Image 2	Image 3	Image 4
CV [8]	Iterations	40	40	40	40
	CPU time(sec)	3.635	3.412	3.245	3.785
LSACM [23]	Iterations	300	300	300	300
	CPU time(sec)	30.458	31.754	31.785	31.254
Bai et al. [27]	Iterations	60	60	60	60
	CPU time(sec)	5.954	5.652	5.742	5.214
VLSBCS. [2]	Iterations	100	100	100	100
	CPU time(sec)	7.324	7.214	6.142	6.475
GLFIF. [30]	Iterations	100	100	100	100
	CPU time(sec)	6.785	8.354	7.024	5.745
HLFRA. [31]	Iterations	100	100	100	100
	CPU time(sec)	6.364	6.705	6.325	5.011
Proposed method	Iterations	50	50	50	50
	CPU time(sec)	**2.748**	**2.877**	**2.923**	**2.821**

To ensure a fair qualitative analysis, we present in Table 5 an asymptotic analysis alongside a comparison with alternative approaches. It is evident from the table that the proposed method shares a cubic time complexity (*O*(*n*^3^)) with some of the existing methods, like LSACM and HLFRA but it has been designed to deliver specific and intended results that might not be achievable with methods that have a lower time complexity. The choice of the appropriate algorithm depends on the specific requirements of the task and the trade-offs between time complexity and the quality of results. In this context, the proposed method has been shown to achieve its intended results effectively, even with its cubic time complexity, making it a valuable addition to the available techniques for the given problem.

**Table 5:** Asymptotic analysis (Big O) previous methods and proposed method.

Methods	Asymptotic Analysis (Big O)
CV [8]	*n* ^2^
LSACM [23]	*n* ^3^
Bai et al. [27]	*n* ^3^
VLSBCS. [2]	*n* ^2^
GLFIF. [30]	*n* ^2^
HLFRA. [31]	*n* ^3^
Proposed method	*n* ^3^

## Quantitative analysis

**Fig 8:**
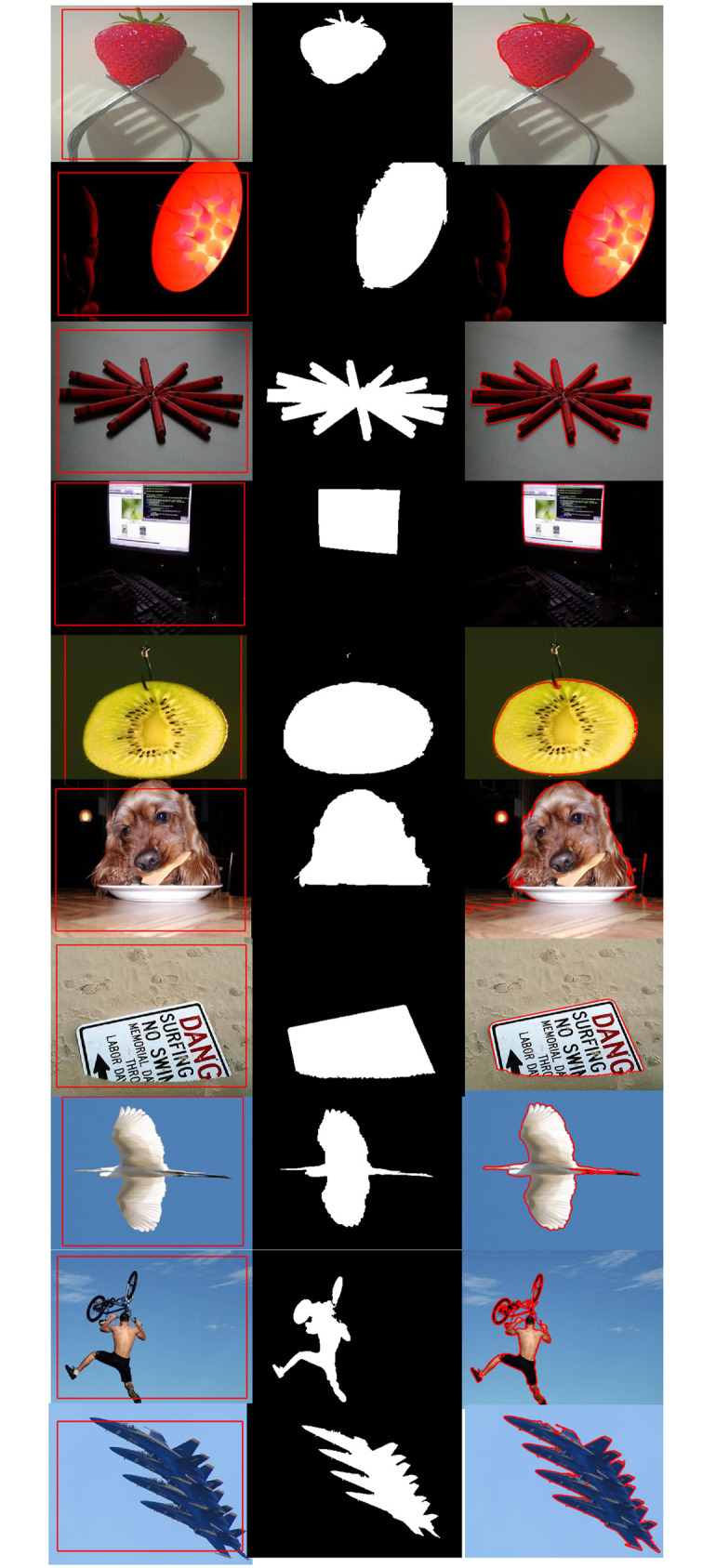
Proposed method validation over [36] dataset. Column 1^*st*^: (Initial Position), Column 2^*nd*^: (Ground truth, 3^*rd*^: (Proposed method result).

For the quantitative analysis of the proposed method. We have taken 100 images with sizes 1024 X1024 from the publicly available dataset [36] and compared the proposed method results with previous methods. Some of the results are illustrated in Fig 8 with the initial position of the contour shown in column I, ground truth is shown in Column II and their segmentation result is displayed in column III.

For quantitative analysis, we use the Dice index, accuracy, specificity, Jaccard index and F1 score metrics. As per definitions of these metrics, the obtained result will be viewed as satisfactory when their values are near 1. The dice index estimates how much perceived cancer matches the ground truth. The accuracy metric alludes to the closeness of the segmented region to its ground truth region. Specificity is how precisely the framework keeps away from true negative regions. Jaccard index coefficient estimates the cross-over of the result over the ground truth and F1 score is the harmonic mean of the precision and recall. The formulation of these metrics is elaborated as below:

$$\begin{array}{c} DSC=\frac{2\times TP}{2\times TP+FP+FN} \end{array}$$ (17)

$$\begin{array}{c} Accuracy=\frac{TP+TN}{TP+TN+FP+FN}, \end{array}$$ (18)

$$\begin{array}{c} Specificity=\frac{\left(TN\right)}{\left(TN+FP\right)} \end{array}$$ (19)

$$\begin{array}{c} Jaccard=\frac{\left(TP\right)}{\left(FP+TP+FN\right)} \end{array}$$ (20)

$$\begin{array}{c} F_{1}=\frac{\left(2TP\right)}{\left(2TP+FP+FN\right)} \end{array}$$ (21)

where, TP (true positive) exhibits segmented cancer tissues, TN (true negative) describes precisely unsegmented tissues, FP (false positive) describes wrongly segmented tissues considered as tumor or region of interest and FN (false negative) describes unsegmented tumor or region.

The quantitative analysis of [36] is illustrated in Table 6 and it shows that proposed formulation has achieved better Dice index, accuracy, specificity and Jaccard index values in comparision with previous formulations.

**Table 6:** Quantitative analysis of the proposed method over [36] dataset.

Methods	DSC	Accuracy	Specificity	Jaccard	F1 Score
CV [8]	0.6475	0.6874	0.6354	0.5934	0.5934
LSACM [23]	0.5247	0.5014	0.5742	0.4845	0.4845
Bai et al. [27]	0.6141	0.5374	0.6274	0.6785	0.6785
VLSBCS. [2]	0.5748	0.5725	0.5654	0.5478	0.5478
GLFIF. [30]	0.7214	0.735	0.7901	0.714	0.7905
HLFRA. [31]	0.7722	0.8014	0.7325	0.7974	0.81145
Proposed method	**0.9014**	**0.896**	**0.8654**	**0.8745**	**0.8829**

**Fig 9:**
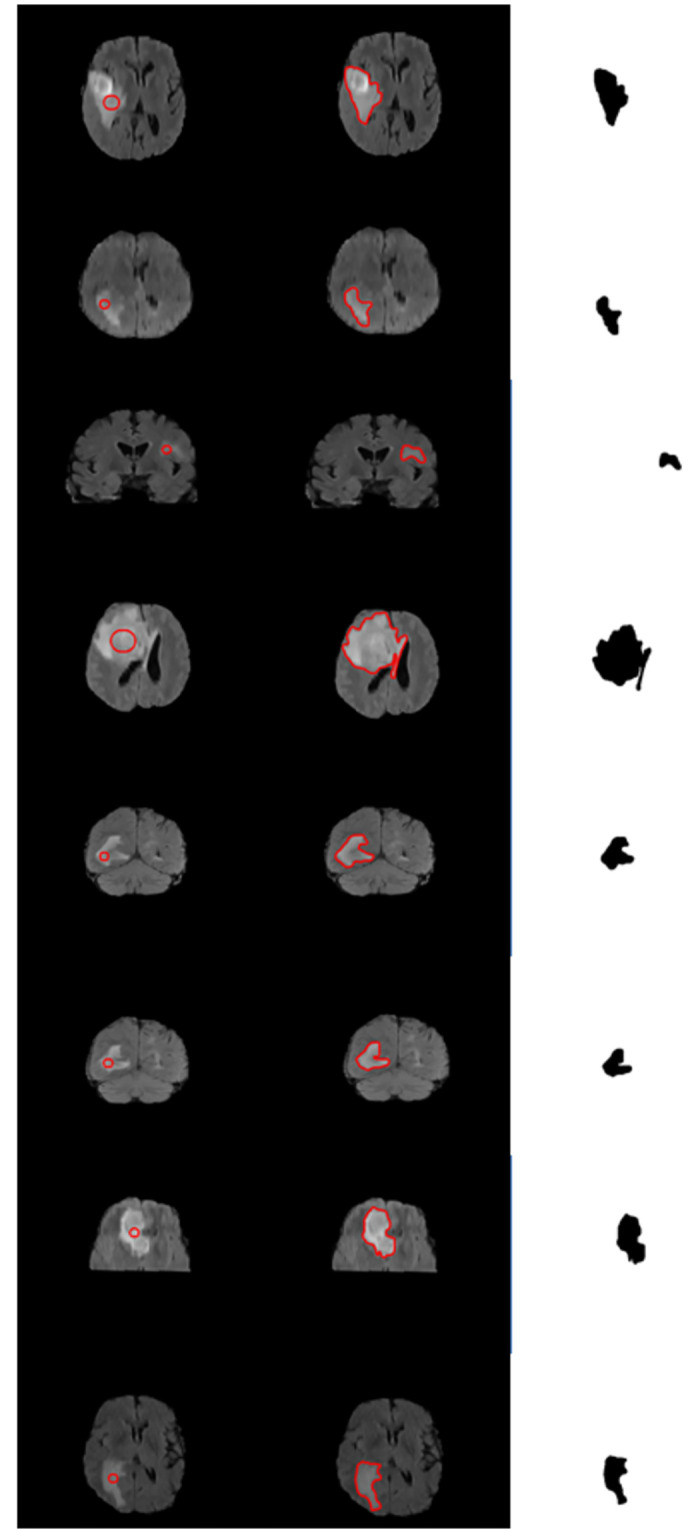
Proposed method validation over BRATS 2015 [33] dataset. Column I: (Initial Position), Column II: (Proposed method segmentation, Column III: (extracted tumor).

Similarly, we have validated the proposed method quantitatively over BRATS 2015 [33] for brain tumor detection. This dataset contains MRI data of four successions for every case known as T1-weighted(T1), T1 with gadolinium-upgrading contrast (T1c), T2-weighted (T2), and FLAIR. More recently, active contours have been adapted frequently by researchers for brain tumor detection [37]. In this regard, we executed our method using BRATS 2015 [33] dataset over 200 HGG(high-grade glioma) and 44 LGG (low-grade) patient volumes. Some of the results of brain tumor detection are demonstrated in Fig 9, where first column illustrates the initial position of the contour, second column shows tumor segmentation and third column shows segmented tumor from an image. Similarly, quantitative results are shown in Table 7. The results show that proposed method have achieved better outcomes for DSC, accuracy, specificity, Jaccard index and F1 score compared to previous methods.

**Table 7:** Quantitative analysis of proposed method over BRATS 2015 [33] dataset.

Methods	DSC	Accuracy	Specificity	Jaccard	F1 Score
CV [8]	0.4365	0.4745	0.4856	0.4856	0.5425
LSACM [23]	0.4954	0.5412	0.4958	0.5245	0.5367
Bai et al. [27]	0.5652	0.5745	0.5845	0.5412	0.5901
VLSBCS. [2]	0.6145	0.6325	0.6214	0.6854	0.6763
GLFIF. [30]	0.8542	0.7415	0.8365	0.7941	0.7458
HLFRA. [31]	0.8012	0.7745	0.8254	0.7436	0.7941
Proposed method	**0.9124**	**0.9052**	**0.8775**	**0.8935**	**0.8936**

## Discussion

### Parameter selection

In this section, we outline the specific parameters utilized in our proposed method to achieve accurate and effective results. The following parameters are discussed in detail:

*ϵ*
  The parameter *ϵ* is employed in both the Dirac delta and Heaviside step functions. It serves the purpose of introducing a finite width or regularization to these functions, rendering them suitable for practical calculations and numerical simulations. Modifying the value of *ϵ* can lead to discontinuities or computational challenges. For our experiments, a fixed value of *ϵ* = 1.5 was used consistently.
*σ*_1_ *and* *σ*_2_
  *σ*_1_ and *σ*_2_ are integral parameters in the Difference of Gaussians (DoG) filter, a widely adopted technique in image processing. The DoG filter serves various purposes, including image enhancement, edge detection, and feature extraction.
  *σ*_1_ represents the standard deviation of the first Gaussian function applied in the DoG filter. A larger *σ*_1_ corresponds to a wider and smoother Gaussian, preserving low-frequency image information while minimizing noise.
  *σ*_2_ signifies the standard deviation of the second Gaussian function in the DoG filter. Typically, *σ*_2_ is chosen to be larger than *σ*_1_. The choice of these parameters affects the scale at which edges are detected. Smaller values of *σ*_1_ and *σ*_2_ highlight fine details, while larger values emphasize broader features.
*ρ*
  The parameter *ρ* is crucial for contour initialization and depends on the segmentation task’s requirements and object characteristics within the image. Setting *ρ* to 1 establishes an initial contour that is relatively impartial and straightforward. This allows the active contour model to adapt flexibly and move towards object boundaries, accommodating a broader range of potential shapes during the segmentation process.
*w*
  The parameter “w” holds significance within the range of 0 to 1 and plays a pivotal role in balancing image regions and edge information. The behavior of the proposed energy function is contingent on the value of “w.” When “w” approaches 0, the energy function functions primarily as a global segmentation method with an added emphasis on saliency information. Conversely, as “w” approaches 1, the energy function functions as a Zero Crossing Detector (ZCD), transforming the proposed method into an inhomogeneous image segmentation technique. This parameter is manually adjusted depending on the image’s intensity levels.
*s*
  The parameter “s” is defined within the range of 0 to 1 and plays a crucial role in assessing the impact of both intensity and saliency terms based on the image’s characteristics. A larger value of “s” prioritizes the intensity term, making it the dominant factor, which is useful for capturing both homogeneous and inhomogeneous images with minimal emphasis on saliency information. A smaller value of “s” shifts the balance in favor of saliency, ideal for highlighting and extracting textual features from the image. The selection of the “s” parameter is made manually, tailored to the specific image type under consideration.

By clearly defining and explaining these parameters, we aim to provide a better understanding of how they are utilized in our proposed method to achieve the desired results.

### Comparision with machine learning based segmentation techniques

Contemporary segmentation techniques have incorporated machine learning, particularly deep learning architectures, to enhance object segmentation [38] due to their remarkable accuracy and resilience. Nonetheless, the adoption of deep learning methods comes with significant resource demands and prerequisites, such as access to ample computational resources and prior data knowledge. In contrast, the proposed approach relies on a level set-based partial differential structure [39], offering advantages that include not necessitating GPUs, substantial physical resources, or any prior familiarity with the data.

### Limitations of the proposed method

Proposed hybrid active contour model, which combines Difference of Gaussians, Chan-Vese global terms, and saliency information, has a few limitations. It relies on manual parameter tuning, making it less automated, and can be computationally intensive. It’s also sensitive to variations in image quality and may require significant computational resources. Additionally, it may not generalize well across different types of images and might demand more manual intervention compared to recent, more user-friendly methods.

## Conclusion

This research has proposed a novel selective homogeneous and inhomogeneous object segmentation driven by an image global SPF (signed pressure force), a difference of Gaussian (DoG) and saliency functions. With the hybrid combination of DoG, Saliency and SPF we have formulated an extraordinary force function, which has disposed of the restrictions of previous level set based methods. Further, the proposed method has expanded its proficiency by including a selective segmentation behavior, which allows us to segment desired region or the region of interest from any image. In the end, we have adjusted the Gaussian filter to regularize the level set and to maintain a strategic distance from a costly reinitialization. We performed several experiments with real and synthetic images for the validation of the proposed method. Moreover, we adapted the Dice index, accuracy, specificity, *F*_1_ score and Jaccard index metrics for the quantitative validation of our method. Results show that the proposed procedure has accomplished improved results that stood out from past methods.

## Supporting information

S1 Data (ZIP)
