# Supplementary figures and images for: Selective image segmentation driven by region, edge and saliency functions
Source: PLoS One. 2023 Dec 15;18(12):e0294789. doi: 10.1371/journal.pone.0294789 (PMC10723724; doi:10.1371/journal.pone.0294789)

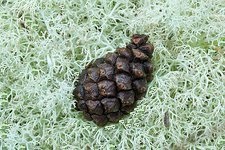

Supplement: S1 Data — (ZIP) [file pone.0294789.s001.zip › Data Availbility/Figure 1/127.jpg]

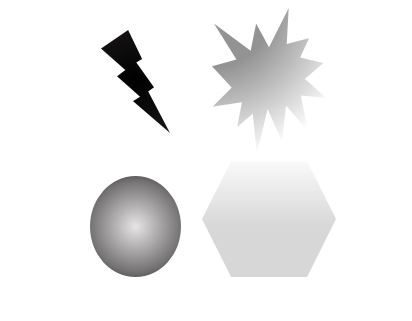

Supplement: S1 Data — (ZIP) [file pone.0294789.s001.zip › Data Availbility/Figure 1/img3.JPG]

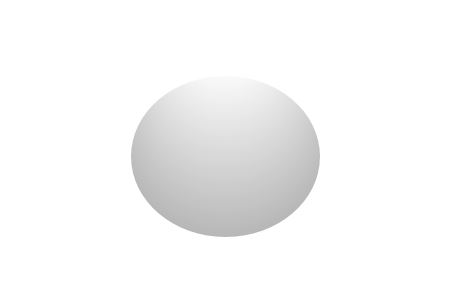

Supplement: S1 Data — (ZIP) [file pone.0294789.s001.zip › Data Availbility/Figure 2 and 4/10.JPG]

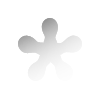

Supplement: S1 Data — (ZIP) [file pone.0294789.s001.zip › Data Availbility/Figure 2 and 4/5.bmp]

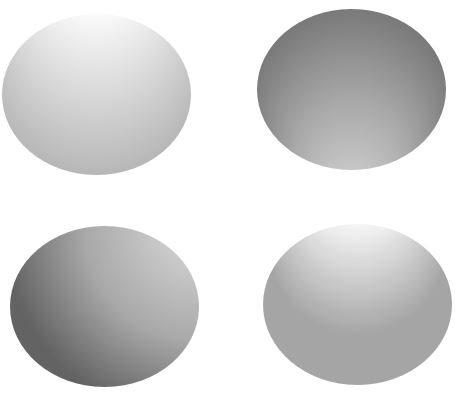

Supplement: S1 Data — (ZIP) [file pone.0294789.s001.zip › Data Availbility/Figure 2 and 4/circle2.JPG]

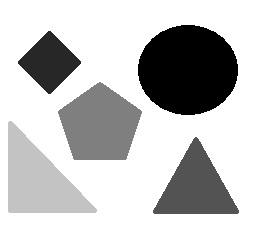

Supplement: S1 Data — (ZIP) [file pone.0294789.s001.zip › Data Availbility/Figure 2 and 4/img7.jpg]

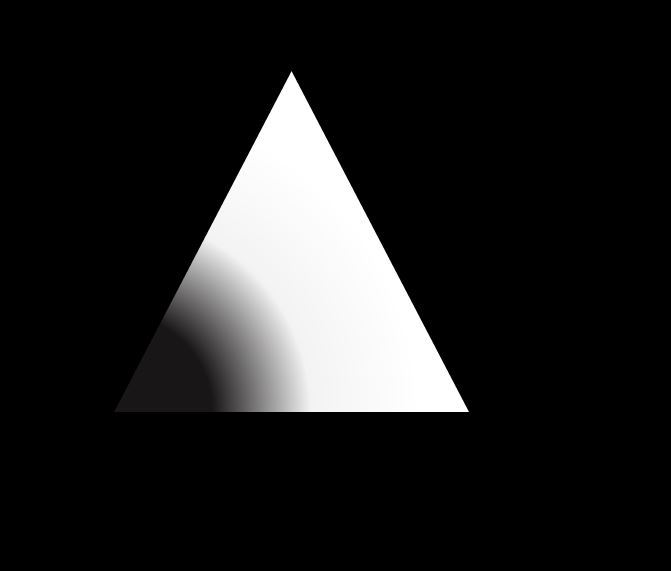

Supplement: S1 Data — (ZIP) [file pone.0294789.s001.zip › Data Availbility/Figure 2 and 4/intense9.JPG]

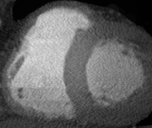

Supplement: S1 Data — (ZIP) [file pone.0294789.s001.zip › Data Availbility/Figure 5/7.bmp]

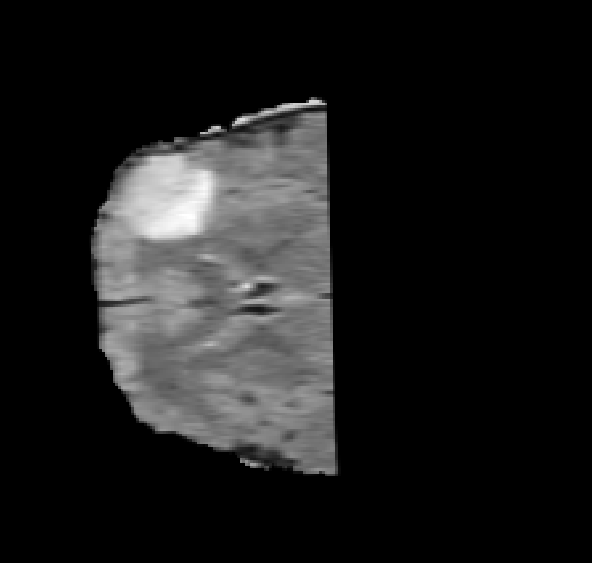

Supplement: S1 Data — (ZIP) [file pone.0294789.s001.zip › Data Availbility/Figure 5/brain13.PNG]

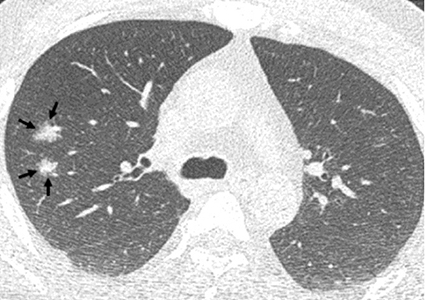

Supplement: S1 Data — (ZIP) [file pone.0294789.s001.zip › Data Availbility/Figure 5/image--618.jpg]

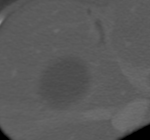

Supplement: S1 Data — (ZIP) [file pone.0294789.s001.zip › Data Availbility/Figure 5/liver_ori.bmp]

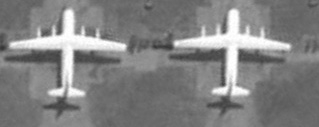

Supplement: S1 Data — (ZIP) [file pone.0294789.s001.zip › Data Availbility/Figure 5/plane2.bmp]

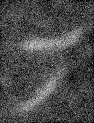

Supplement: S1 Data — (ZIP) [file pone.0294789.s001.zip › Data Availbility/Figure 6/w_0.01.bmp]

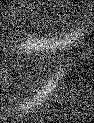

Supplement: S1 Data — (ZIP) [file pone.0294789.s001.zip › Data Availbility/Figure 6/w_0.04.bmp]

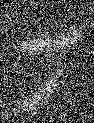

Supplement: S1 Data — (ZIP) [file pone.0294789.s001.zip › Data Availbility/Figure 6/w_0.1.bmp]
